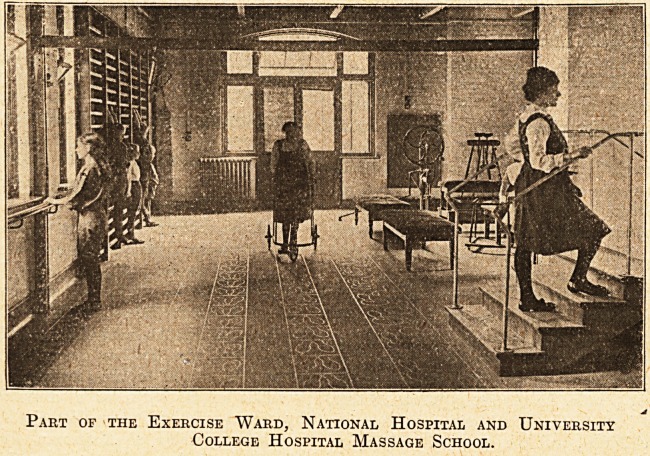# The Combined Scheme of Two Well-Known Hospitals

**Published:** 1917-10-06

**Authors:** 


### TRAINING IN MASSAGE.

### The Combined Scheme of Two Well-known Hospitals.

JOINT enterprise on the part of hospitals has been tried in many directions, such as laundry schemes, x-ray work, and training of nurses. We believe, however, that the first example of a combined scheme in respect of training in massage, etc., is the new School of Massage, Electrical Treatment, and Swedish Remedial Exercises, which has been recently founded under the joint management of University College Hospital and the National Hospital for the Paralysed and Epileptic.

It is obvious in the case of the National Hospital, where the nursing training is of a specialised character, that in order to attract nurses some branch of teaching not always obtained elsewhere is a great incentive to women to join the nursing staff. In respect of University College Hospital, as may be said of all general hospitals, the more complete the education of the nurses the more useful they are in their service to the hospital, and the greater will be the credit to their training-school through the work performed by them in their after career. In addition there are facilities at the one hospital which are not possessed by the other, and the combination enables the student to cover the whole field of study. Primarily, therefore, the training of nurses is in each case a sufficient *raison d'être* for the foundation of the school.

### A COMPREHENSIVE CURRICULUM.

The new institution, however, is open to receive approved pupils of both sexes who are not nurses, and in connection with the school there is a well-appointed hostel where the women students can reside during term-time. The curriculum embraces instruction in massage, remedial exercises, medical electricity, hydrotherapeutics, x-ray and light treatment. It is understood, however, that the last three subjects will not be undertaken until the expiry of the war, this decision being caused rather by the lack of teachers than of special departments in which practical education can be given. Part of the instruction in anatomy will be given by members of the medical staff of University College Hospital, and the physician in charge of the electrical department of the National Hospital will lecture to the students of the school of medical electricity. The students will receive their instruction in the school and in the wards and outpatient and special departments of the two hospitals. The up-to-date character of the arrangements at University College Hospital are well known to most of our readers, and at the National Hospital there is a department, the exercise ward, greatly developed during the last two or three years, which has been found indispensable in the treatment of many cases both on the military and civil sides of the hospital. As is well known, in many forms of nervous diseases persons lose the power of carrying out complicated movements such as walking, and have to learn over again how to use their limbs. The ward, of which we give an illustration, contains all that is necessary for the teaching of Swedish remedial exercises, and is equipped with machines invented by Dr. Zander for the strengthening of limbs and breaking down of adhesions of joints.

PART OF THE EXERCISE WARD, NATIONAL HOSPITAL AND UNIVERSITY COLLEGE HOSPITAL MASSAGE SCHOOL.

The school, which is situated in two commodious houses adjoining the National Hospital, contains a large classroom, recreation and reading rooms for the students, and rooms for practical demonstration in massage. Miss Eileen Peel, the recently appointed head, was trained at the Swedish Institute of Massage in Cromwell Road, and holds the teachers' certificate of the Incorporated Society of Trained Masseuses. Miss Peel has held the post of sister-in-charge of the massage, electrical, and x-ray department at the North Staffordshire Infirmary, Stoke-on-Trent, and has since had a wide experience as instructress in several London schools. The first term of six months' massage teaching commences on October 15. The electrical treatment, with a term of three months, opens on October 16, and the Swedish remedial exercises commence in January next, a course of twenty-four weeks' instruction. The fees are as follows: Massage course, £15 15s.; remedial exercises, £15 15s.; medical electricity, £5 5s. Full particulars of the school can be obtained from the secretary at 29 and 30 Queen Square.

## Figures and Tables

**Figure f1:**